# Environmentally Friendly Cross-Linked Antifouling Coatings Based on Dual Antimicrobial Action

**DOI:** 10.3390/ijms22094658

**Published:** 2021-04-28

**Authors:** Georgia C. Lainioti, Anthi Tsapikouni, Denisa Druvari, Pavlos Avramidis, Ioannis Prevedouros, Alexios Glaropoulos, Joannis K. Kallitsis

**Affiliations:** 1 Department of Chemistry, University of Patras, GR–26504 Patras, Achaia, Greece; up1053327@upatras.gr (A.T.); druvari@upatras.gr (D.D.); kallitsi@upatras.gr (J.K.K.); 2 Department of Geology, University of Patras, GR–26504 Patras, Achaia, Greece; p.avramidis@upatras.gr (P.A.); ioannispreve@gmail.com (I.P.); 3 AVRAMAR EU, Old National road Patras, Athens 55, 26500 Rio, Achaia, Greece; a.glaropoulos@avramar.eu

**Keywords:** environmentally friendly antimicrobial polymers, cross-linking reaction, antifouling efficiency, quaternary ammonium compounds, aquaculture nets, coating

## Abstract

The synthesis of environmentally friendly antimicrobial polymeric coatings, especially in the case of aquaculture, that inhibit the growth of bio-deposits is a very important issue that will contribute to the cost reduction of nets’ cleaning process as well as the protection of the submarine wealth from the biostatic substances used so far. In the present work, the antimicrobial polymers P(SSAmC_16_-co-VBCHAMx) and the terpolymer P(SSAmC_16_w-co-VBCHAMx-co-GMAy) were synthesized, bearing quaternary ammonium compounds, electrostatically bound and covalently attached at the same polymer chain. The combination of the two types is of particular importance, as it can provide effective antimicrobial polymeric materials with self-polishing capabilities as a result of the released nature of the antimicrobial, in combination with the permanent local action of the immobilized species. The cross-linking reaction of the terpolymer P(SSAmC_16_w-co-VBCHAMx-co-GMAy) with the homopolymer polyacrylic acid (PAA) was tested at 120 °C in terms of the equivalent ratio between epoxy and carboxyl groups. The synthesized polymers were further used for the coating of aquaculture nets and tested in terms of antifouling efficiency in lab and scale-up conditions. Uncoated nets were also used in all applications for comparison reasons. The coated nets performed efficiently for 35 days in lab-scale and 66 days in scale-up conditions, showing a high antifouling activity in both fields compared to the uncoated nets.

## 1. Introduction

The design of environmentally friendly antimicrobial polymers is part of an important research field posing challenges, such as metal-free technologies that inhibit the growth of microorganisms (fungi/algae/parasites) on ship reefs or fishing nets (biofouling) [1,2,3]. Especially in the case of fish farming, biofouling is a critical economic and environmental problem, as it leads to reduced efficiency and increased cleaning costs and often harms the ecosystem due to the biostatic substances used so far.

The most common methodology for preventing or delaying biofouling involves the use of antifouling coatings/paints [4,5]. In current technology, copper is usually the main biocidal component, whereas zinc in the form of zinc pyrithione may also be applied [6,7]. As a result, high concentrations of copper are often observed in sediments near fish farms, which may affect fish mortality and also lead to bioaccumulation of minerals in fish tissues [8,9].

To address various problems in antifouling applications in several fields, intense research activity has been developed in the direction of alternative biostatic materials, environmentally acceptable, with improved or multiple functions. Antimicrobial polymers describe an attractive suggestion in the field of antimicrobial coatings and membranes [10,11,12], as they lack the disadvantages of low-molecular-weight antimicrobials (such as volatility, environmental toxicity, short duration of action, and human or animal skin penetration), while maintaining the advantages of their polymeric nature (ability to form membranes and coatings, various synthetic methods, and flexibility in chemical methods of modification).

Thus, antimicrobial polymers are a rapidly growing field, as a number of research activities have been published in recent years, with a variety of systems proposed for many applications [13,14]. In most systems, the antimicrobial activity is owed to the incorporation of groups based on quaternary ammonium salts with a long alkyl chain into the polymer. The action of these materials depends on the adsorption of the positively charged part of the active compound from the negatively charged surface of the bacterial cell and the subsequent penetration through the cell wall and the lipophilic aliphatic chain, which eventually leads to cell death.

Depending on the way of binding onto the polymeric chain, antimicrobial groups can be divided into two categories: immobilized, where there is a covalent bond of the groups to the polymer, and released [15,16,17], where antimicrobial groups are electrostatically bound on the polymer chain. The combination of the two types is of particular importance, as it may lead to effective antimicrobial polymeric materials with self-polishing abilities, as a result of the released nature of the one kind of antimicrobial groups, but also permanent action, owed to the immobilized species. Moreover, taking into account that these materials may be incorporated into polymeric matrices, it is possible to control the release not only of the antimicrobial group but also of the polymer in general.

In this line, the members of our research team are dynamically involved in the development of copolymers bearing immobilized and released antimicrobial groups at the same polymer network. The antimicrobial moieties that are used as immobilized and released species are 4-vinylbenzyldimethylhexadecylammonium chloride (VBCHAM) and 4-styrenesulfonate cetyltrimethylammonium (SSAm_16_), [18]. The statistical copolymers [19] as well as the diblock copolymers [20], which have been synthesized by our group, comprise the abovementioned moieties and demonstrate the significant antibacterial activity of suitable polymeric structures against Gram-negative and Gram-positive bacteria.

In a more complex version, cross-linked polymeric systems have also been developed in which the two kind of antimicrobial groups coexist [21,22], utilizing the central idea of reactive blending. This methodology enables the reaction of mixtures of two copolymers having complementary chemically active groups, such as epoxy and carboxyl groups. Under this perspective, cross-linked polymeric membranes were prepared by our group after active mixing of statistical copolymers, combining the two types of antimicrobial binding species, VBCHAM and SSAmC_16_ groups, as well as functional groups such as carboxylic groups of acrylic acid (AA) and epoxy groups of glycidyl methacrylate (GMA).

In the present work, aiming at a dual action, the copolymers P(SSAmC_16_-co-VBCHAMx), bearing both released and immobilized antimicrobial groups in a statistical architecture, were developed. Taking advantage of the proven antimicrobial activity of the abovementioned copolymers, the latest idea in a recent development was formulated and the antimicrobial terpolymer P(SSAmC_16_w-co-VBCHAMy-co-GMAx) was synthesized, bearing also chemical groups so that they can react after heat treatment. The cross-linking reaction between the epoxide groups and acrylic acid allowed the preparation of mechanically stable films and coatings with antimicrobial action. In this way, the long-term action and the controlled release of the antimicrobial component were achieved at the same time, enabling the development of coatings with very good properties that can be used for various applications. The abovementioned coatings were studied in terms of their antifouling efficiency, in accelerated conditions, as well as in a scale-up process, for the coating of aquaculture nets, which were subsequently submerged in a fish farm unit.

## 2. Results and Discussion

In the present work, the copolymers P(SSAmC_16_-co-VBCHAM30) and P(SSAmC_16_-co-VBCHAM70) and the terpolymer P(SSAmC_16_60-co-VBCHAM20-co-GMA20) were selected in order to be applied as antifouling coatings on aquaculture nets. All the abovementioned polymeric structures bore electrostatically bound biocidal species (cetyltrimethylammonium cations, AmC_16_) as well as immobilized biocidal species (*N,N*-dimethylhexadecylamine, HAM) at the same polymeric chain. The idea was conceived by our laboratory team and was thoroughly studied in order to create antifouling coatings that are environmentally friendly and effective for a long period of time. Moreover, the terpolymer P(SSAmC_16_60-co-VBCHAM20-co-GMA20) was combined with the homopolymer PAA in order to be applied as net coating. Under this perspective, the system contained not only electrostatically bound and immobilized antimicrobial groups but also epoxide functional groups (glycidyl methacrylate, GMA) and acrylic acid functional groups (acrylic acid, AA), which subsequently reacted, providing a cross-linked coating.

### 2.1. Synthesis and Characterization of Polymers

**Figure 1:**
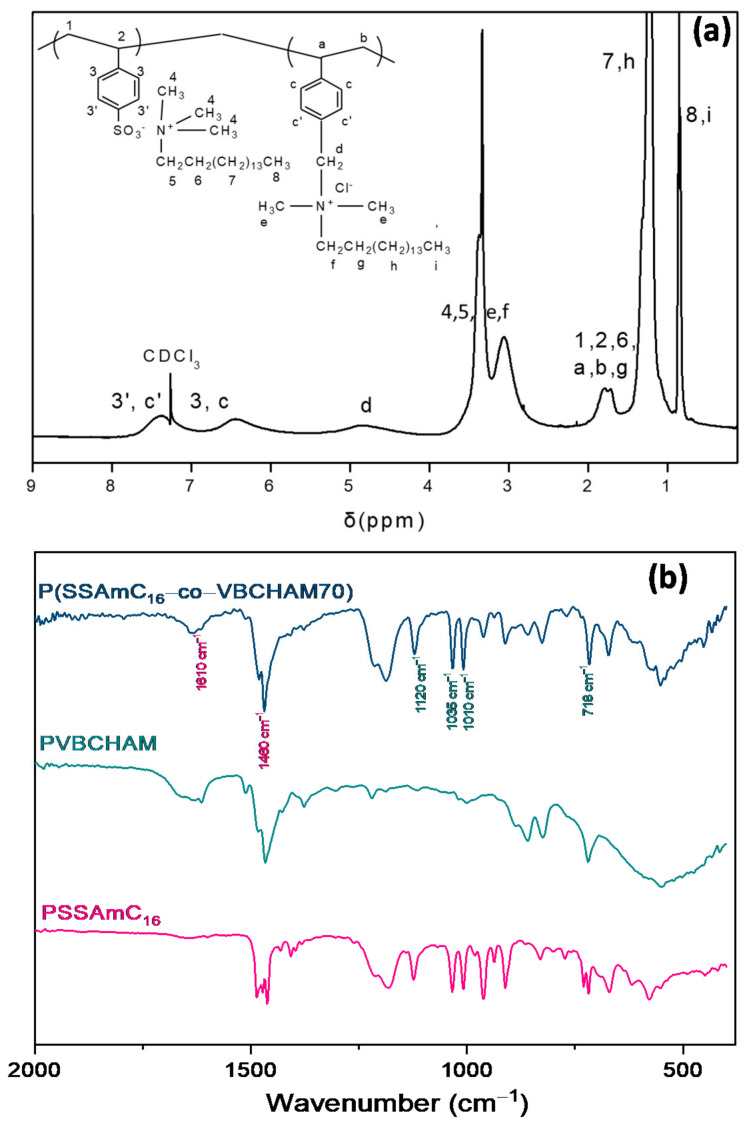
(**a**) The ^1^H-NMR and (**b**) ATR-FTIR spectra of the copolymer P(SSAmC_16_-co-VBCHAM70).

The copolymers P(SSAmC_16_-co-VBCHAM30) and P(SSAmC_16_-co-VBCHAM70) contain electrostatically bound (cetyltrimethylammonium cations, AmC_16_) and immobilized antimicrobial species (*N,N*-dimethylhexadecylamine, HAM). The characterization of the synthesized copolymers was verified through Proton Nuclear Magnetic Resonance (^1^H NMR) in deuterated chloroform (CDCl_3_) and Attenuated Total Reflection Fourier Transform Infrared Spectroscopy (ATR-FTIR) spectroscopy. The characteristic spectrum of P(SSAmC_16_-co-VBCHAM70) is shown in Figure 1a.

The protons of the main chain of the copolymer (1, 2, a, b) were observed in the range 1.6–1.8 ppm, while the peaks at 6.5–7.5 ppm corresponded to the protons of the aromatic rings of both structural units (3, 3 ‘, c, c ‘). The protons of CH_3_ and CH_2_ groups (4, 5, e, f) linked with the nitrogen atoms were found at 3.06 and 3.3 ppm. The existence of VBCHAM was also confirmed from the broad peak at 4.8 ppm (d), attributed to the protons linked with the quaternary nitrogen atom (CH_2_N^+^). Moreover, at 0.86 ppm and 1.28 ppm we observed the protons of the CH_3_ groups (8, i) and the 13 CH_2_ groups (7, h), respectively. The remaining CH_2_ groups (6, g) appeared between 1.6–1.8 ppm with the protons of the main chain, as mentioned above.

The synthesized copolymers were also characterized by ATR-FTIR spectroscopy. The spectrum of P(SSAmC_16_-co-VBCHAM70) is presented in Figure 1b. Spectra of the homolymers PSSAmC_16_ and PVBCHAM are also shown, for comparison reasons. The peaks at 1460–1486 cm^−1^ corresponded to the CH_2_ peaks of the PSSAmC_16_ and PVBCHAM homopolymers and the N–CH_3_ mode [23,24]. In the spectrum of the statistical copolymer, the peaks of PVBCHAM were observed to prevail, confirming its high composition in it. The peaks at 1010 cm^−1^ and 1035 cm^−1^ were due to the symmetrical vibration, while the peak at 1120 cm^−1^ was due to the asymmetric vibration of the SO^3−^ units. The peak at 718 cm^−1^ was also related to the CH_2_ peaks. The absorption peaks of VBCHAM at 1610 cm^−1^ and 1460 cm^−1^ were attributed to the C=C bond of the aromatic ring and the C-N bond, respectively.

During the ^1^H NMR characterization of the homopolymer PAA the protons of the main chain of the homopolymer appeared in the range 1.4–1.9 ppm, while at 2.3–2.5 ppm the acidic proton was observed (data not shown).

**Figure 2:**
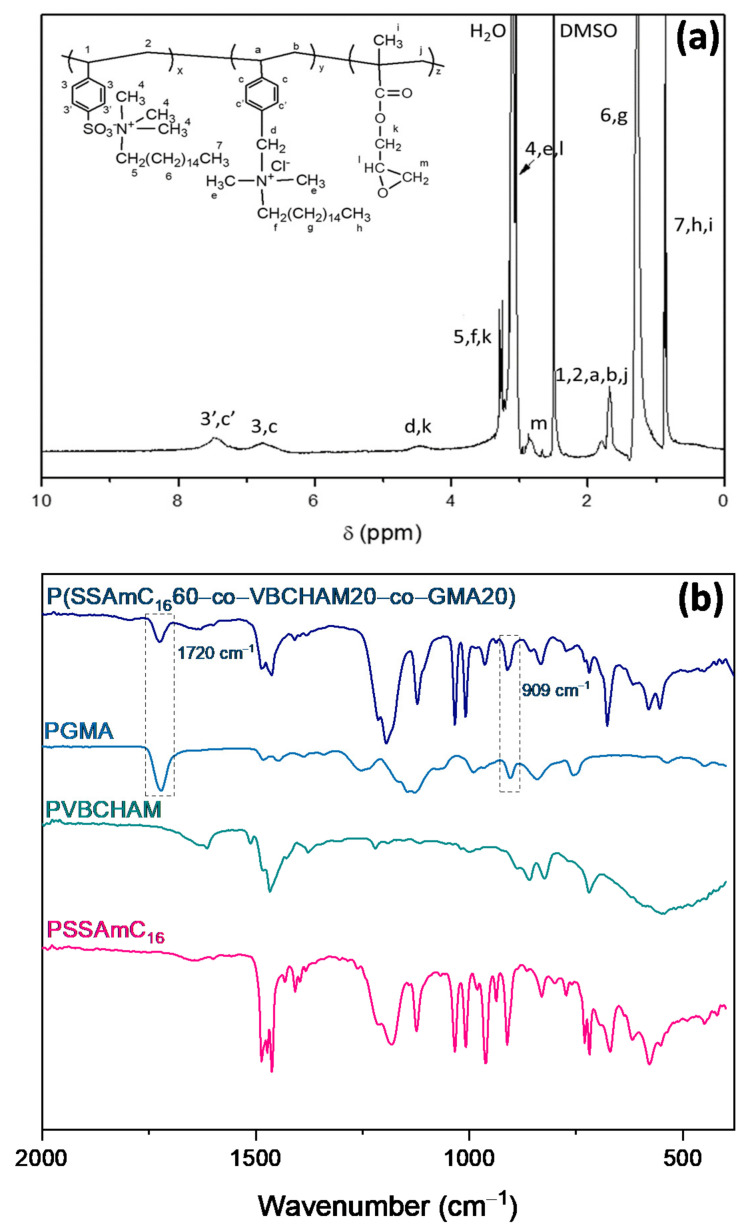
(**a**) The ^1^H-NMR and (**b**) ATR-FTIR spectra of the terpolymer P(SSAmC_16_-co-VBCHAMx-co-GMAy).

The synthesis of the terpolymer P(SSAmC_16_60-co-VBCHAM20-co-GMA20) was verified through the ^1^H-NMR spectra, shown in Figure 2a. The protons from the main chain of the copolymer (1, 2, a, b, j) were observed in the range of 1.6–1.8 ppm, while the peaks at 6.5–7.5 ppm corresponded to the protons of the aromatic rings (3, 3 ‘, c, c’). The protons of the methyl groups attached to the nitrogen atoms (4, e) appeared at 3.3 ppm, while the protons of the final methyl groups of the amines (7, h), as well as the methylene group (i), appeared at 0.8 ppm. Finally, methylene groups (5, f) and (6, g) were observed at 3.2 and 1.3 ppm, respectively. Except for the characteristic peaks of the SSAmC_16_ and VBCHAM moieties that were described above, characteristic peaks of GMA were also observed in the spectra.

The ATR-FTIR spectra of P(SSAmC_16_-co-VBCHAMx-co-GMAy) and the respective homopolymers PSSAmC_16_, PVBCHAM, and PGMA are shown in Figure 2b. The introduction of PSSAmC_16_ and PVBCHAM were verified from the characteristic peaks described above, whereas the introduction of PGMA units was strongly confirmed by the peak at 1720 cm^−1,^ which was attributed to the carbonyl stretch of C=O group, and the peak at 909 cm^−1^ of the epoxy group.

### 2.2. Optimization of the Cross-Linking Reaction

As mentioned above, the terpolymer P(SSAmC_16_60-co-VBCHAM20-co-GMA20), which has both covalently and electrostatically linked antimicrobial groups as well as the active epoxy group, was combined with the homopolymer PAA, which carries carboxyl groups, in order to create a cross-linked coating, which appeared to improve stability during application on the aquaculture nets. In an attempt to optimize the cross-linked coating, different compositions of the two complementary copolymers were investigated, while the content of epoxy groups (GMA) in the terpolymer remained 20 mol%. Thus, mixtures of the complementary polymers were prepared in different compositions using 95% ethanol as solvent, which are shown in Table 1. Membranes were then obtained through solvent casting at room temperature. The cross-linking reaction allowed us to proceed in the solid state at 120 °C for 24 h. Several pairs of the copolymers were investigated in order to cover different mixing ratios, *r*, of functional units (*r* = nGMA/nAA), where n GMA and n AA are the equivalents of GMA and AA units, respectively, ranging from *r* = 10/1 (GMA-rich mixtures) up to *r* = 0.3/1 (AA-rich mixtures).

**Figure 3:**
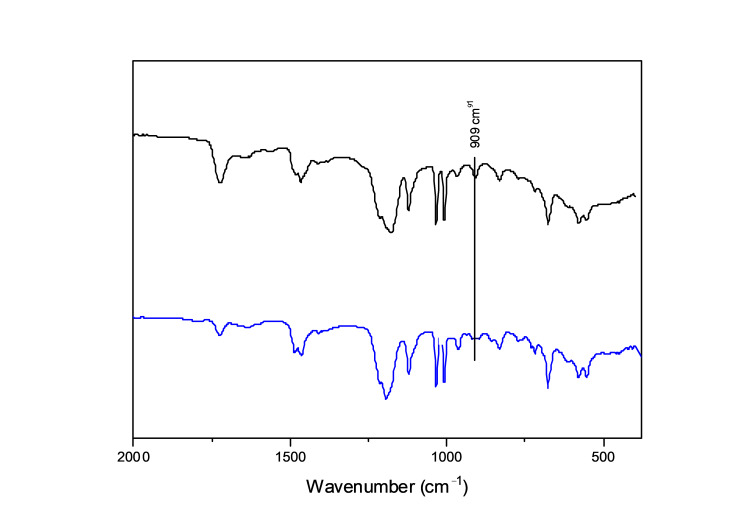
ATR-FTIR spectra of the film P(SSAmC_16_60-co-VBCHAM20-co-GMA20)/PAA before (black line) and after (blue line) cross-linking at 120 °C.

The success of the cross-linking reaction was observed through ATR-FTIR characterization of the synthesized membranes. Figure 3 presents the spectra of the film created between P(SSAmC_16_60-co-VBCHAM20-co-GMA20) and PAA before and after cross-linking at 120 °C. The characteristic peaks that were examined in order to verify the cross-linking reaction were the peak at 1720 cm^−1,^ which corresponded to the carbonyl stretch of C=O group, and the peak at 909 cm^−1,^ which was a representative peak of the epoxy group. As observed in the spectra of the cross-linked film, the peak at 909 cm^−1^ diminished to a great extent, showing the ring opening of GMA and, therefore, the reaction between epoxy and carboxyl groups.

As the cross-linking reaction had affection for the membranes’ solubility, the films were immersed in 95% ethanol and NaCl 0.8 M for seven days. The solubility studies are quoted in Table 1. The concentration of the NaCl solution simulated the salinity conditions of seawater, while ethanol was selected as it was the solvent used for membranes’ formation. In some cases, the results gave supporting evidence of the success of the cross-linking reaction between epoxy and carboxyl groups of the complementary polymers. More specifically, membranes M-85 and M-95 were insoluble not only in ethanol but in NaCl as well. The only difference was that M-95 became soft after seven days of immersion in NaCl, in contrast to M-85, which was maintained in the initial situation. This is a very important result for the final application, as a more rigid and stable material is preferable for a scale-up coating performance. The rest of the membranes were either soluble or partially soluble in the ethanol or NaCl aqueous solution.

The abovementioned observations led to the selection of the system P(SSAmC_16_60-co-VBCHAM20-co-GMA20)/PAA in composition 85/15 (% wt) for the final application as coating on aquaculture nets at accelerated and scale-up conditions.

### 2.3. Net Coating for Laboratory Accelerated Conditions

The polymers P(SSAmC_16_-co-VBCHAM30) and P(SSAmC_16_-co-VBCHAM70) were dissolved in CHCl_3_. Moreover, the polymer blend P(SSAmC_16_60-co-VBCHAM20-co-GMA20)/PAA in composition 85/15 (% wt) was dissolved in ethanol 95%, as previously described. Pre-weighed nets cut into dimensions of 20 × 25 cm were immersed in the blend solutions and left for 1 h. Nets were subsequently left to dry at Room Temperature (R.T.) and then cured at 120 °C, for stabilization of the coating and cross-linking in the case of the complementary polymers. The polymer uptake of the nets was between 25–32% (*w/w*), as shown in Table 2.

The coated nets, as well as an uncoated net (blank) for comparison reasons, were immersed in glass tanks filled with seawater and remained for 35 days. In order to accomplish accelerated conditions of algae growth, the tanks were filled with seawater containing 3.5 mL of a Walne medium nutrient solution and 3.5 mL of algae aliquot. Tanks were also equipped with four multispectral lamps with a 500 lux of illuminance on the top [25]. As the algal growth cycle lasts for six to seven days, the seawater nutrient solution was renewed on the seventh day and the experiment was conducted for another week. The same procedure took place at the 14th and the 21st days.

**Figure 4:**
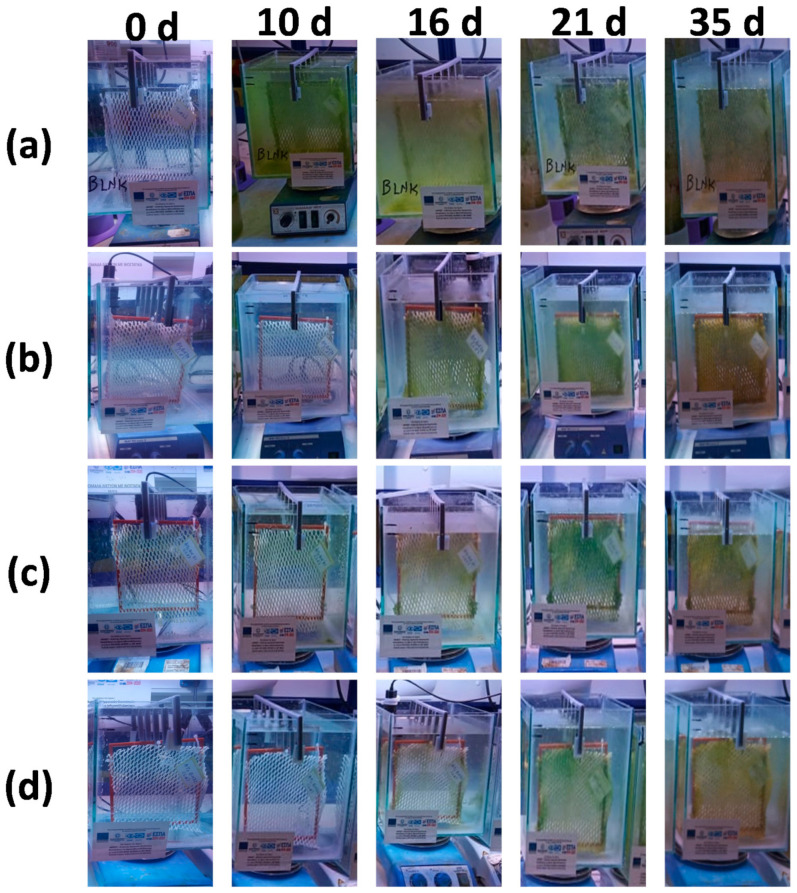
Photographs of the uncoated (**a**) and the coated (**b**–**d**) nets at the beginning of the immersion (t = 0 d) and after 10 d, 16 d, 21 d, and 35 d of immersion.

The immersed nets were optically observed periodically during the experimental procedure, and photographs at 0, 10, 16, 21, and 35 days are shown in Figure 4.

The photographs clearly show the difference between the uncoated and the coated nets, as the uncoated net (blank) presented a high-fouling behavior accompanied by turbidity of the seawater solution even at the 10th day. The nets (b) and (c) coated with the copolymers P(SSAmC_16_-co-VBCHAM30) and P(SSAmC_16_-co-VBCHAM70), as well as the net (d) coated with the blend P(SSAmC_16_60-co-VBCHAM20-co-GMA20)/PAA in composition 85/15 (% wt), showed similar behavior, with net (b) performing slightly better showing low algal growth and seawater solution turbidity even after 35 days of immersion. From the abovementioned results, we concluded that the combination of quaternary ammonium compounds, electrostatically bound (SSAmC_16_) and covalently attached (VBCHAM) at the same polymer chain, ensured the longer activity of the coated nets in contrast to the uncoated net. This behavior was obviously owed to the simultaneous existence of a release-killing and a direct contact-killing effect deriving from the electrostatically attached and the immobilized ammonium compounds, respectively. This observation agrees with literature where a contact-killing mechanism has been described by numerous studies, indicating that antimicrobial polymers may inhibit bacterial proliferation through contact with microorganisms’ cell membrane, causing its disruption and consequently cell death [26,27]. Still, another way of action of antimicrobial polymers, called release-killing effect, was responsible for the leakage of the electrostatically bound moieties into the boundary layer forming an inhibition zone [28,29].

### 2.4. Net Coating for Scale-Up Application

In order to test the effectiveness of new materials in the field, the copolymers P(SSAmC_16_-co-VBCHAM30) and P(SSAmC_16_60-co-VBCHAM20-co-GMA20) were synthesized in larger quantities. Nets with dimensions of 30 × 20 cm were pre-dried and used for the coating procedure.

To achieve the best cross-linking behavior and high antimicrobial efficiency, a blend of the terpolymer P(SSAmC_16_60-co-VBCHAM20-co-GMA20) with PAA was prepared in the concentration 85/15 (% wt), which gave the most promising results in the aforementioned laboratory test. The coating percentage ranged between 35–40%. The coated and uncoated nets (blank nets) were mounted onto an aquaculture cage and immersed in the sea for 25 days.

**Figure 5:**
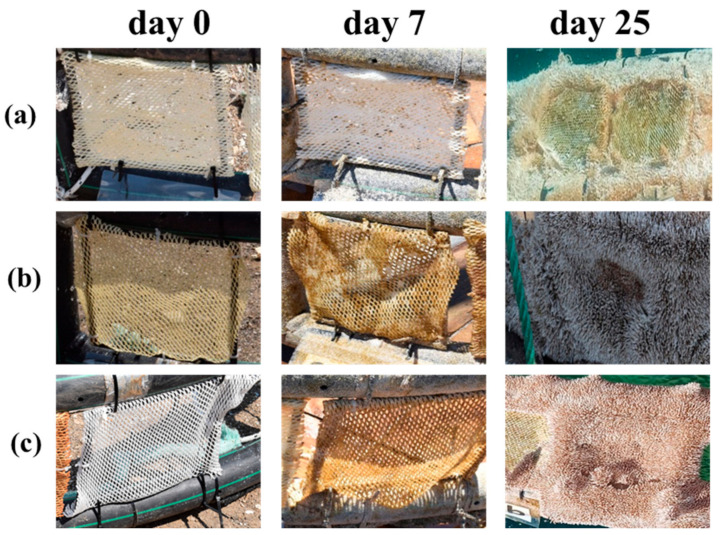
Photographs of the nets coated with P(SSAmC_16_-co-VBCHAM30) (**a**), P(SSAmC_16_60-co-VBCHAM20-co-GMA20)/PAA (**b**), and uncoated net (**c**) at the beginning of the immersion (t = 0 d) and after 7 d and 25 d of immersion in the sea environment.

Photographs of the nets are presented in Figure 5.

As it clearly may be seen, blank nets showed high fouling at the 10th day of immersion whereas the coated nets seemed to be more resistant. The difference between the coated and uncoated nets was more obvious at the 25th day of immersion, where blank nets were totally covered by fouling organisms and the coated nets still had intact areas. It is important to note that the immersion of the aquaculture cage with the nets was conducted during summer, a period with biofouling pressure. This is an important observation as, according to Bloecher et al., (2013) [30], nets are washed every eight weeks in winter and every two weeks in summer, but it may occur as often as weekly due to the enhanced growth of fouling organisms in warmer months. Moreover, when mild temperatures occur in the sea environment (5–20 °C), biofouling arises during the entire year, showing strong seasonality from spring (beginning of April) to early autumn (end of October), when the most spawning and growth of microorganisms takes place [31,32]. Depth and light availability have also a great effect on the composition and growth of biofouling organisms. Macrofoulers, such as macroalgae, which are photosynthetic organisms, are usually more abundant in sections within the euphotic zone (0–40 m), which is characterized by higher temperatures and light and high levels of plankton [33,34].

Moreover, as it may be seen from Figure 5, fouling microorganisms started to grow at the cage and then transferred to nets, which were tightly fastened onto the cage. After these observations, our next thought was that this phenomenon could be avoided if nets had a greater distance from the aquaculture cage. Moreover, a slight movement of nets could simulate to a better level the real conditions of an aquaculture cage, which is moved either from the wave intensity or from the fish inside it.

**Figure 6:**
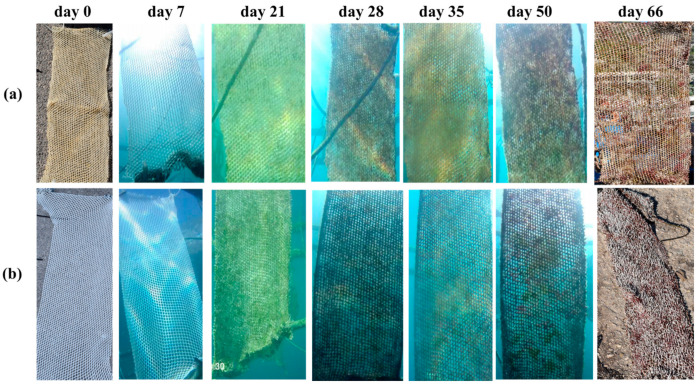
Photographs of the nets coated with P(SSAmC_16_-co-VBCHAM70) (**a**) and uncoated net (**b**) at the beginning of the immersion (t = 0 d) and after 7 d, 21 d, 28 d, 35 d, 50 d, and 66 d of immersion in the sea environment.

Thus, in order to minimize the problems of high-fouling pressure, we made further tests with longer net pieces with dimensions of 0.5 m (width) × 1 m (length), which were placed on the aquaculture cage with the longer part to hang in order to be free and have the ability to move in the direction of sea currents. On the bottom of the nets, weights were placed to protect nets and keep them in a vertical position. The abovementioned conditions simulate to a great extent the way of preparation and setting of the aquaculture cages into the sea environment of an aquaculture unit. The system selected for these field tests was the copolymer P(SSAmC_16_-co-VBCHAM70), which showed the best results in the accelerated conditions. The coating percentage was 40%. Photographs of the coated and uncoated nets at various time intervals between the pilot-scale tests are presented at Figure 6. As it may be seen, the net coated with the antimicrobial polymer exhibited higher antifouling efficiency than the uncoated (blank) net. The differences were more obvious especially after the 21st day of immersion, when the uncoated net showed high fouling and the antimicrobial nets had still unaffected areas (distinguished as white spots). 

**Figure 7:**
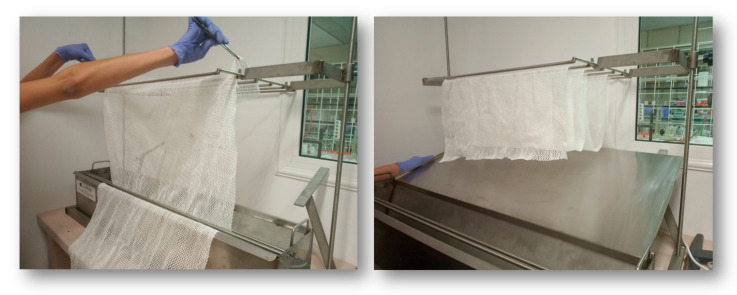
Homemade bath for nets’ coating with antimicrobial polymers on a pilot scale, equipped with a drying hang and a stainless-steel inclined surface.

For the pilot-scale coating of antimicrobial polymers on the aquaculture nets, the homemade bath was used, equipped with a drying system (Figure 7), as described in the Experimental section.

Impressive was the behavior of nets at the end of the scale-up test (66th day), when the uncoated net showed a high fouling with microorganisms grown on it, in contrast to coated nets, which showed milder fouling. The coated net combined the effectiveness of the release-killing effect of SSAmC_16_ units with the contact-killing action of the VBCHAM moieties. Thus, the low-fouling behavior of the coated net was obviously the result of the combination of the release-killing effect coming from the electrostatically attached ammonium compounds SSAmC_16_ with a direct contact-killing effect deriving from the immobilized ammonium compounds of VBCHAM. However, the contact-killing type had a significant weakness related to the antibacterial action that may be limited to the surface, reducing the effectiveness of the coating when foulants will cover the surface [35]. Thus, the combination of electrostatically attached and immobilized ammonium compounds at the same polymer chain enhanced the coating antifouling efficiency by providing it with a two-fold functionality, deriving from the release-killing and the contact-killing effect.

It is also worth mentioning that antifouling coatings have been evaluated mostly through field experiments on ship hulls or aquaculture nets. As stated by the Efficacy Assessment Guideline of the European Chemical Agency (ECHA), field experiments are required in order to estimate antifouling efficiency in natural conditions where biofouling takes place [36]. In this way, the common technique to assess the antifouling efficiency of coatings is the wait-and-see approach, which includes the immersion of coated panels or nets by hanging them on test panels and detecting biofouling. In tests conducted in aquaculture units in sea environments, conditions of the marine environment, like seasonal variations, depth, and light availability, have a great effect on biofouling test results [37]. However, because field experiments usually need varying numbers of coated panels or nets and a long time to obtain results, making them overpriced to perform, the conduction of experiments under controlled laboratory conditions is appropriate in order to assess antifouling coatings. Therefore, in efficiency tests of antifouling coatings, it is important to establish a controlled experimental condition, either in laboratory or in field tests in order to ensure reproducible results.

In a further stage, UV crosslinked functional groups could be incorporated in the polymeric chains and the cross-linking reaction could be tested in terms of curing time. We may well test this idea in our next steps since many parameters have to be examined in order to achieve the polymer functionalization with the UV cross-linked functional groups and the optimization of the cross-linking reaction.

## 3. Materials and Methods

### 3.1. Materials

The monomers glycidyl methacrylate (GMA), 4-vinylbenzyl chloride (VBC), acrylic acid (AA); the amine *N,N*-dimethylhexadecylamine (HAM); the initiators azobisisobutyronitrile (AIBN), potassium persulfate (KPS), and potassium disulfite (PDS); and the surfactant cetyltrimethylammonium bromide (CTAB), as well as deuterated chloroform (CDCl_3_), were purchased from Aldrich (Aldrich, Steinheim, Germany) and used as received. The monomer sodium 4-styrene sulfonate (SSNa) was purchased from TOSOH Europe B.V. (TOSOH Europe B.V., Amsterdam, Netherlands) and used as received. The solvents dimethylformamide (DMF), chloroform (CHCl_3_), diethyl ether, dimethyl ether, and ethyl acetate were purchased from Fischer Scientific (Fisher Scientific, Pittsburgh, PA, USA) and used as received without further purification. Ultra-pure water was obtained by means of an SG apparatus water purification unit. Nylon nets used for coating were purchased from HelNet S.A., Schimatari, Greece.

A Walne medium nutrient solution was used in order to achieve accelerated algae growth during the experiments. Walne’s medium components and concentrations were given in De Martino et al., 2007 [38]. In addition, algae culture was obtained using the standard cultivation process described by Laing, 1991 [25].

### 3.2. Synthesis of Quaternized SSAmC_16_ and VBCHAM

The quaternization of the monomer SSNa took place through an ion exchange reaction in aqueous solution between the sodium ions of SSNa units with an excess of quaternary ammonium cations of CTAB. A precipitation obtained after the end of the reaction was held at (R.T.). The final product, denoted as SSAmC_16_, was obtained after filtration, washing with ultrapure H_2_O, and drying under vacuum at 60 °C for 24 h.

For the quaternization of VBC with HAM, a solution of VBC and HAM in DMF was stirred at room temperature for 72 h. The final product, denoted as VBCHAM, was recovered after precipitation in ethyl acetate, filtered, washed with hot hexane, and dried under vacuum at 60 °C for 24 h [18].

### 3.3. Synthesis of the Copolymers P(SSAmC_16_-co-VBCHAMx)

The copolymers, 4-styrenesulfonate cetyltrimethylammonium-vinyl benzyl dimethylhexadecylammonium chloride, were synthesized through free radical copolymerization in CHCl_3_ using AIBN as initiator. The experimental procedure was previously described [39]. Briefly, the desired quantities of the monomers SSAmC_16_ and VBCHAM (total monomer concentration 1 M) were dissolved in a 500-mL, round-bottom flask equipped with a reflux condenser and dissolved in CHCl_3_. The solution was degassed and the initiator AIBN (0.8 mol% over the total monomer concentration) was added. The reaction was left to proceed overnight under vigorous stirring in Ar atmosphere in an oil bath set at 70 °C. The next day, after cooling down to room temperature, the copolymer was recovered by precipitation in ethyl acetate, filtered, and dried in a vacuum oven at 60 °C for 24 h. The mol fraction of SSAmC_16_ and VBCHAM was calculated from ^1^H NMR spectroscopy using CDCl_3_ as solvent. The copolymers are denoted as P(SSAmC_16_-co-VBCHAMx) where x is the mol fraction of VBCHAM units. The copolymers synthesized in the present work were P(SSAmC_16_-co-VBCHAM30) and P(SSAmC_16_-co-VBCHAM70). ATR-FTIR spectroscopy was also conducted for the characterization of the copolymers P(SSAmC_16_-co-VBCHAMx).

### 3.4. Synthesis of the Terpolymer P(SSAmC_16_w-co-VBCHAMx-co-GMAy)

The terpolymer was synthesized through free radical copolymerization using AIBN as initiator. More specifically, in a 500-mL, round-bottom flask equipped with a reflux condenser, the desired quantities of the monomers SSAmC_16_, VBCHAM, and GMA (total monomer concentration 1 M) were dissolved in DMF. The solution was degassed and the initiator AIBN (0.8 mol% over the total monomer concentration) was added. The reaction was left to proceed overnight under vigorous stirring in Ar atmosphere in an oil bath set at 80 °C. The final product was obtained after precipitation in dimethyl ether, filtration, and drying at 40 °C in a vacuum oven. The final terpolymer is denoted as P(SSAmC_16_w-co-VBCHAMx-co-GMAy) where w, x, and y are the mol fractions of SSAmC_16_, VBCHAM, and GMA units, respectively. The terpolymer synthesized in the present work was P(SSAmC_16_60-co-VBCHAM20-co-GMA20). ATR-FTIR spectroscopy was also conducted for the characterization of the synthesized terpolymer.

### 3.5. Synthesis of Polyacrylic Acid (PAA)

A typical procedure of aqueous solution polymerization was followed for the synthesis of polyacrylic acid. A certain amount of NaOH was dissolved in deionized water and then acrylic acid was added and stirred by magnetic stirrer in a 200-mL flask at room temperature (pH: 4–7). Afterwards, a mixture of initiators, potassium persulfate and potassium disulfite, each dissolved in deionized water separately, were added to the monomer solution and the flask was deoxygenated by bubbling argon through the solution for 30 min. Then, it was immersed into an oil bath at 65 °C and the polymerization was allowed to proceed for 24 h. Almost immediately, the reaction mixture viscosity increased significantly, indicating the polymer formation. The polymer solution was placed in a dialysis membrane (MWCO: 12,000) and, finally, the product was obtained through freeze-drying. The homopolymer was characterized through ^1^H-NMR and ATR-FTIR spectroscopies and its molecular weight was determined through Size Exclusion Chromatography (Mw: 220,000).

### 3.6. Cross-Linking Reaction

After the synthesis of the antimicrobial terpolymer P(SSAmC_16_w-co-VBCHAMx-co-GMAy), the cross-linking reaction with the homopolymer (PAA) was thoroughly tested. Specifically, each polymer was dissolved in ethanol until obtaining homogenous solutions. Then, the two solutions were mixed and the final blend solution was used as coating for aquaculture nets.

In order to find the optimum cross-linking conditions, various parameters were studied, such as blend composition, the solvent, the temperature of cross-linking, the time of curing, and the equivalents of GMA and AA. The tested parameters were analyzed and shown in Table 1 in the Section 2.

### 3.7. Solubility Studies

Small pieces of the cross-linked pre-weighed films were placed in glass vials containing ethanol, the solvent used for the film creation, and left at room temperature for 48 h. Visual observations took place initially. Then, films were removed from ethanol solutions, dried, and weighed in order to check the weight losses.

### 3.8. Coating of Aquaculture Nets with Antimicrobial Systems

The copolymers P(SSAmC_16_-co-VBCHAM30) and P(SSAmC_16_-co-VBCHAM70) were dissolved in CHCl_3_ at 10% (*w/v*) concentration. After complete dissolution, pre-weighed nets were immersed in the solution and left for a while. The nets were initially left to dry at R.T. and then were placed in an oven at 80 °C for complete drying and stabilization of the coating onto the net. The polymer uptake of the nets was 35% (*w/w*).

For net coating with the antimicrobial system P(SSAmC_16_60-co-VBCHAM20-co-GMA20)/PAA, each polymer was dissolved in ethanol at 10% (*w/v*) concentration. The two solutions were then mixed and a pre-weighed net was immersed into the solution and left for a while. Following the same procedure with the copolymers mentioned above, the net was dried at R.T. and then cured at 120 °C for cross-linking. The polymer uptake of the nets was 25% (*w/w*).

### 3.9. Antifouling Assessment in Lab-Scale Accelerated Conditions

The coated nets were placed onto polymeric frames and immersed into different glass aquariums filled with seawater, 3.5 mL of a Walne medium nutrient solution, and 3.5 mL of algae aliquot in order to achieve 1.0 mL of algae–nutrient mixture per liter of seawater. An uncoated net (blank) was also added in one aquarium as the control sample for comparison reasons. Four multispectral lamps with a total of 500 lux of illuminance were placed on the top of the aquariums. The seawater temperature in the aquariums ranged from 18.4–29.7 °C, the pH from 8.17 to 9.88, O_2_ from 5.63 to 16.88 ppm, and salinity from 39.7 to 41.0 g/kg. The accelerating eutrophication conditions at the uncoated net (blank) were examined daily through optical observation, in order to confirm that adequate repeatable conditions were obtained on the fourth day of each experiment initiation.

### 3.10. Pilot-Scale Coating of Antimicrobial Polymers on Aquaculture Nets

The copolymers P(SSAmC_16_-co-VBCHAM30) and P(SSAmC_16_-co-VBCHAM70) were dissolved in CHCl_3_ at 10% (*w/v*) concentration. The terpolymer P(SSAmC_16_60-co-VBCHAM20-co-GMA20) and the homopolymer PAA were dissolved in ethanol at 10% (*w/v*) concentration, as described above. After blend preparation, pre-weighed, dried nets with dimensions of 30 cm (width) × 20 cm (length) were immersed into the coating solutions. The nets were left to dry at R.T. and then cured at 80 or 120 °C. In a further step, larger nets were also used with dimensions of 0.5 m (width) × 1 m (length), which were also immersed into the coating solution. The coating procedure was carried out in a specially designed homemade bath, as shown in Figure 7. The bath was made from stainless steel and had length of 1.5 m. It was equipped with a scroll bar, in order to remove the unnecessary material, and a rack for net draining. Under the rack, a stainless-steel surface was placed with inclination in order to ensure that the unnecessary coating material returned to the bath. Nets were handled on the rack for 30 min in order to dry initially at room temperature (R.T.). The coating was repeated until the polymer uptake of the nets ranged between 30–40% (*w/w*). The nets were then placed in an oven at 80 or 120 °C for complete drying and cross-linking per case. Then the coated nets were weighed in order to determine the exact coating uptake. After the completion of the coating procedure, nets were transferred in an aquaculture unit. There, the nets were hooked on a specially designed aquaculture cage and immersed in the Saronic Bay of Greece. For comparison reasons, blank nets were also immersed into the sea. All the nets were placed in the same depth to obtain constant lighting and ventilation conditions. Photographs of the nets were obtained at regular time intervals in order to control the fouling effect.

### 3.11. Chemical Characterization

#### 3.11.1. Proton Nuclear Magnetic Resonance (^1^H-NMR)

The samples for ^1^H-NMR characterization were prepared by dissolving the copolymers P(SSAmC_16_-co-VBCHAMx) and P(SSAmC_16_w-co-VBCHAMx-co-GMAy) in CDCl_3_ and DMSO, respectively. The ^1^H-NMR spectra were obtained at 400 MHz at 300 K on a Bruker AVANCE DPX 400 spectrometer (Bruker BioSpin GmbH, Magnet Division, Karlsruhe, Germany). The ^1^H-NMR spectra were used to determine the chemical composition of the copolymers.

#### 3.11.2. Attenuated Total Reflection Fourier Transform Infrared Spectroscopy (ATR-FTIR)

The ATR-FTIR spectra of the copolymers and cross-linked films before and after curing were recorded using a Bruker Optic’s Alpha-P Diamond ATR Spectrometer of Bruker Optics GmbH (Ettlingen, Germany).

## 4. Conclusions

The research focused on the development of dual action copolymers P(SSAmC_16_-co-VBCHAMx), bearing both released and immobilized antimicrobial groups, in a statistical architecture. The proven antimicrobial activity of those polymers led to the synthesis of the novel antimicrobial terpolymer P(SSAmC_16_-co-VBCHAMy-co-GMAx), bearing not only the two kinds of antimicrobial groups but also chemical groups so that they can react after heat treatment. The cross-linking reaction between the epoxide groups of the terpolymer and the acrylic acid groups of the homopolymer PAA allowed the preparation of a novel polymeric coating, mechanically stable with antimicrobial action.

The abovementioned systems were tested in terms of their antifouling efficiency in accelerated laboratory conditions as well as in field test applications. Moreover, the antifouling efficiency of all the coated nets was higher than the uncoated (blank) net, which, at the end of the experiments, was completely covered by fouling microorganisms. On the contrary, nets coated with the antimicrobial polymers exhibited high-fouling efficiency with unaffected areas observed even at the end of the immersion. Thus, all the polymeric systems described in the present work could be efficient coatings with application in the aquaculture sector. However, the results indicated a slightly better performance for the nets coated with the copolymer P(SSAmC_16_-co-VBCHAM30).

## Figures and Tables

**Table 1:** Solubility tests of P(SSAmC_16_60-co-VBCHAM20-co-GMA20)/PAA membranes after curing at 120 °C.

P(SSAmC_16_60-co-VBCHAM20-co- GMA20)/PAA %(*w/w*)	*r* (nGMA/ nAA)	Solubility Tests in Ethanol (*) (1% *w/v*)	Solubility Tests in NaCl 0.8 M (*)	Membrane Code
95/5	10/1	insoluble	insoluble (soft)	M-95
85/15	3/1	insoluble	insoluble	M-85
75/25	2/1	insoluble	partially soluble	M-75
55/45	0.7/1	soluble	insoluble	M-55
35/65	0.3/1	soluble	partially soluble	M-35

(*): Optical observation after the seventh day.

**Table 2:** Aquaculture nets, blank and coated with antimicrobial polymeric coatings.

Aquarium	Code	Equivalents’ Ratio	Curing Temperature (°C)	Net Coating (%)
1	Blank	-	-	-
2	P(SSAmC_16_-co-VBCHAM30)	-	120	31
3	P(SSAmC_16_-co-VBCHAM70)	-	120	32
4	P(SSAmC_16_60-co-VBCHAM20-co-GMA20)/PAA	3/1	120	25

## Data Availability

No new data were created or analyzed in this study. Data sharing is not applicable to this article.
